# A step forward in addressing cancer survivorship in the Asia-Pacific region

**DOI:** 10.1186/s12916-017-0796-6

**Published:** 2017-01-26

**Authors:** Raymond Javan Chan, Alexandre Chan, Patsy Yates, Alex Molassiotis

**Affiliations:** 1 School of Nursing and Institute of Health and Biomedical Innovation, Queensland University of Technology, Brisbane, Australia; 2 Cancer Nursing Professorial Precinct, Royal Brisbane and Women’s Hospital, Brisbane, Australia; 3 Department of Pharmacy, Faculty of Science, National University of Singapore, Singapore, Singapore; 4 Department of Pharmacy, National Cancer Centre, Singapore, Singapore; 5 School of Nursing, Hong Kong Polytechnic University, Kowloon, Hong Kong SAR China; 6 Level 3, School of Nursing, N Block, Queensland University of Technology - Kelvin Grove Campus, Kelvin Grove, Q4059 Australia

**Keywords:** Asia, Asia-Pacific region, Cancer survivorship, Cancer policy, Service planning, Low- and middle-income countries

## Abstract

Cancer survivorship is being increasingly recognized as an important component of cancer care. This commentary reviews the key findings reported in the recent *BMC Medicine* publication of the ACTION study, which focuses on the health-related quality of life and psychological distress in 5249 cancer survivors in eight low- and middle-income countries in Southeast Asia. The study identified that more than one-third of survivors experience at least mild levels of anxiety and depressive symptoms and that poorer outcomes in quality of life, anxiety, and depressive symptoms are linked to a number of clinical and demographic factors. Such data provides an important foundation to inform cancer policy and service planning in Asia. Future research efforts are required to further understand the needs of cancer survivors in this region and determine interventions to improve outcomes for this population.

Please see related article: http://bmcmedicine.biomedcentral.com/articles/10.1186/s12916-016-0768-2.

## Background

Cancer incidence in the Asia-Pacific region accounts for over 30% of the global worldwide cancer burden [1]. Ongoing efforts to improve cancer services in the areas of prevention, early detection and screening, and treatment, have resulted in a growing population of cancer survivors across the region [2]. Despite major advances in anti-cancer treatment, improved outcomes in terms of cure or control of the disease do not necessarily guarantee a full restoration of health, with many survivors continuing to experience impaired quality-of-life as a result of the disease or its treatments. Therefore, cancer survivorship is being increasingly recognized as an important part of cancer care. There has been limited research to date focusing on understanding the health needs of cancer survivors and improving outcomes for this population, especially in the Asia-Pacific region.

## An important step to inform care and policy

In a recent article in *BMC Medicine*, Kimman et al. [3] seek to gain an understanding of quality of life and psychological distress in Asian cancer survivors at 12 months post-diagnosis. The authors measured quality of life, anxiety, and depressive symptoms in 5249 survivors in eight low- to middle-income countries (LMICs) using the European Organisation for Research and Treatment of Cancer Quality of Life Questionnaire (EORTC QLQ-C30), the EuroQol-5 dimensions questionnaire, and the Hospital Anxiety and Depression Scale. Where a formally validated translated tool was not available, the authors used the forward-translation and backward-translation World Health Organization guidelines [4]. Kimman et al. [3] should be commended for completing this large-scale international study. Undertaking international studies is often resource intensive, requiring collaboration with many investigators and stakeholders, numerous ethics and local approvals, and protocolized translation of research instruments.

In addition to documenting the significant health-related quality of life (HRQoL) and psychological needs of cancer survivors in the Asia region, the Kimman et al. [3] study identifies specific groups who are at risk of poorer outcomes. That is, the authors identified that patients with lung cancer and lymphoma had lower level of global HRQoL as well as higher prevalence of self-reported anxiety and depressive symptoms. This finding is not surprising due to a number of known factors that are associated with increased distress, including the poorer prognosis and greater symptom burden associated with lung cancer and the intense nature of treatment of lymphoma. The authors also highlight a number of other factors significantly associated with a lower level of quality of life and higher levels of anxiety and depressive symptoms, including being older, being male, having an advanced stage at diagnosis, being of low-income status, and not being in paid work. Such findings have potential implications for health services and clinicians in their prioritization of care and resources.

This pioneering study provides an important foundation for future work in this region. We make some additional observations for consideration by those with an interest in advancing cancer services for this population. Firstly, HRQoL and psychological distress are key patient-reported outcomes that should be an important focus for cancer control efforts. A better understanding of physical symptom burden of patients beyond those measured by the EORTC QLQ-C30 is also required. Moreover, most available data focus on cancer survivors in the western countries. Future studies could allow for benchmarking and comparisons between western countries that have more developed survivorship service delivery models, high-income Asian countries, and Asian LMICs. Such comparisons would allow for meaningful benchmarking exercises that can eventually inform service planning.

Although the study provides useful preliminary findings, the authors themselves acknowledge a number of key limitations that should be taken into account when interpreting the results. Of the limitations mentioned by the authors, one of the most critical is the lack of data collection and lack of reporting of change scores over time between diagnosis and at 12 months. Predictive relationships should only be drawn on a true empirical basis with the use of longitudinal data [5]. While the authors provided a rationale for only including data at 12 months, each patient’s quality of life and experience are uniquely influenced by a number of factors including their treatment plans. We argue that to provide a more comprehensive understanding of the multiple influences on health-related outcomes, it is important to understand changes in outcomes over time taking into account differences in treatment regimens and pathways.

The study involved combining data from all eight countries for analysis. We believe that additional insights could also be gained if between-country differences were reported. There is considerable variation between countries in health systems, culture, and resources. Therefore, understanding differences in health outcomes for cancer survivors between countries would provide useful data to further examine how health system, cultural, and resource differences contribute to differences in outcomes for cancer survivors.

Kimman et al. [3] provide several useful recommendations concerning future efforts to improve care provision and policy development. In order to translate these data into practice, we suggest the development of consensus statements and recommendations for minimum standards for cancer survivorship care provision for countries in this region. Kimman et al. [3] rightly point out the lack of resources and poor social economic positions of the LMICs in this region. Lack of resources and lack of clarity concerning health professionals’ responsibilities have been repeatedly identified to be key barriers for delivering survivorship interventions [6, 7]. Development of survivorship care plans and treatment summaries is highly resource intensive, requiring skilled health professionals to complete them [7]. It is therefore important that future research examines the appropriateness and potential benefits of survivorship care plans and treatment summaries in these countries.

A number of interventions for improving outcomes in the post-treatment phase have been proposed in the western context [8–11]. These efforts include a range of complex interventions comprising survivorship care plans and follow-up care from designated health professionals. Despite the largely negative findings in the two completed trials [8, 11], it is important to continue efforts to optimize outcomes for cancer survivors through high quality, culturally appropriate and sustainable survivorship care models [12]. A recent pilot randomized controlled trial reported that a culturally adapted psycho-education program was feasible and potentially effective in improving outcomes in Asian breast cancer patients [13]. It is important that governments continue to invest into developing and testing culturally appropriate, evidence-based service models and interventions for patients in this region [12].

## Conclusions

The ACTION study has made an original, important contribution to the field by providing an understanding of HRQoL and psychological distress in Asian cancer survivors. It is important to acknowledge that this study is a starting point, with much future work required to further understand and meet the needs of cancer survivors in the Asia-Pacific region. Cancer survivorship care is a public health issue, requiring system-, organizational-, and professional- level responses. We urge researchers, clinicians, policymakers, and healthcare administrators to invest in future efforts to improve care for the ever-growing population of cancer survivors.
